# Are immune-related adverse events associated with the efficacy of immune checkpoint inhibitors in patients with cancer? A systematic review and meta-analysis

**DOI:** 10.1186/s12916-020-01549-2

**Published:** 2020-04-20

**Authors:** Xiaoxiang Zhou, Zhuoran Yao, Huaxia Yang, Naixin Liang, Xuan Zhang, Fengchun Zhang

**Affiliations:** 1 Department of Rheumatology and Clinical Immunology, Peking Union Medical College Hospital, Chinese Academy of Medical Sciences and Peking Union Medical College, The Ministry of Education Key Laboratory, Beijing, 100730 China; 2 Department of Thoracic Surgery, Peking Union Medical College Hospital, Chinese Academy of Medical Science & Peking Union Medical College, Beijing, 100730 China

**Keywords:** Immune-related adverse events, Immune checkpoint inhibitors, Cancer, Efficacy

## Abstract

**Background:**

A number of studies have reported an association between the occurrence of immune-related adverse events (irAEs) and clinical efficacy in patients undergoing treatment with immune checkpoint inhibitors (ICIs), but the results remain controversial.

**Methods:**

Under the guidance of a predefined protocol and Preferred Reporting Items for Systematic Reviews and Meta-analyses statement, this meta-analysis included cohort studies investigating the association of irAEs and efficacy of ICIs in patients with cancer. The primary outcome was overall survival (OS), and the secondary outcome was progression-free survival (PFS). Subgroup analyses involving the cancer type, class of ICIs, combination therapy, sample size, model, landmark analysis, and approach used to extract the data were performed. Specific analyses of the type and grade of irAEs were also performed.

**Results:**

This meta-analysis included 30 studies including 4971 individuals. Patients with cancer who developed irAEs experienced both an OS benefit and a PFS benefit from ICI therapy compared to patients who did not develop irAEs (OS: hazard ratio (HR), 0.54, 95% confidence interval (CI), 0.45–0.65; *p <* 0.001; PFS: HR, 0.52, 95% CI, 0.44–0.61, *p <* 0.001). Subgroup analyses of the study quality characteristics and cancer types recapitulated these findings. Specific analyses of endocrine irAEs (OS: HR, 0.52, 95% CI, 0.44–0.62, *p <* 0.001), dermatological irAEs (OS: HR, 0.45, 95% CI, 0.35–0.59, *p <* 0.001), and low-grade irAEs (OS: HR, 0.57, 95% CI, 0.43–0.75; *p <* 0.001) yielded similar results. The association between irAE development and a favorable benefit on survival was significant in patients with cancer who were undergoing treatment with programmed cell death-1 inhibitors (OS: HR, 0.51, 95% CI, 0.42–0.62; *p <* 0.001), but not cytotoxic T-lymphocyte antigen-4 inhibitors (OS: HR, 0.89, 95% CI, 0.49–1.61; *p* = 0.706). Additionally, the association was significant in patients with cancer who were treated with ICIs as a monotherapy (OS: HR, 0.53, 95% CI, 0.43–0.65; *p <* 0.001), but not as a combination therapy (OS: HR, 0.62, 95% CI, 0.36–1.05; *p* = 0.073).

**Conclusions:**

The occurrence of irAEs was significantly associated with a better ICI efficacy in patients with cancer, particularly endocrine, dermatological, and low-grade irAEs. Further large-scale prospective studies are warranted to validate our findings.

**Systematic review registration:**

PROSPERO CRD42019129310.

## Background

Immune checkpoint inhibitors (ICIs) targeting cytotoxic T-lymphocyte antigen-4 (CTLA-4) or programmed cell death-1 (PD-1) pathways are reshaping the landscape of cancer therapy, yielding unprecedented clinical success in treating multiple cancer types [1]. By blocking the inhibitory pathway between T lymphocytes and tumor cells or antigen-presenting cells, ICIs aim to release the brake of the anergized T cells and reactivate their antitumor cytolytic function [2]. Monoclonal antibodies targeting CTLA-4 and PD-1/programmed cell death ligand-1 (PD-L1) axes are currently two major categories applied in cancer immunotherapies. Immune-related adverse events (irAEs) are a unique spectrum of side effects of ICIs that resemble autoimmune responses. irAEs affect almost every organ of the body and are most commonly observed in the skin, gastrointestinal tract, lung, and endocrine, musculoskeletal, and other systems [3]. Since irAEs occur via a process of immune activation, suggesting that the exhausted immune cells have been reinvigorated and attack not only tumor cells but also normal tissue, theoretically, the occurrence of irAEs may indicate a better response to ICI therapy. Nevertheless, whether irAE development is predictive of the ICI response remains controversial.

A number of recent studies has supported this hypothesis by showing favorable outcomes for patients with non-small cell lung cancer (NSCLC) and melanoma who developed various irAEs in response to immune checkpoint inhibition [4–22]. However, a definite conclusion has not been drawn based on the findings from each single study, as contradictory results exist [23–35]. A systemic review included 16 studies and reported that irAEs such as pneumonitis, thyroid disorders, myalgias, and mucosal toxicity did not show a significant correlation with overall survival (OS) [36]. However, this review contained an insufficient number of studies and pooled analyses were not performed. Controversy persists regarding whether irAE development predicts the ICI response. A robust and precise systemic review is required to evaluate the association between irAE occurrence and the efficacy of ICIs.

Herein, we conducted a systemic review of the published studies to investigate the association between irAE occurrence and the efficacy of ICIs. We used a standard meta-analysis approach to obtain a statistical and comprehensive view of the association. Our study addressed the question using the PECO tools: (P) patients with cancer receiving ICIs, (E) occurrence of irAEs, (C) non-occurrence of irAEs, (O) efficacy of ICIs (measured using different outcomes). To the best of our knowledge, the present study is the first meta-analysis to explore the association of the occurrence of irAE and the efficacy of ICIs by pooling the results of eligible studies collectively. Additionally, we separately pooled the predictive effects of different irAE types and irAE grades to investigate their specific roles in homogeneous settings.

## Methods

This systematic review and meta-analysis was conducted under the guidelines of the Preferred Reporting Items for Systematic Reviews and Meta-analyses (PRISMA) statement [37]. We prospectively registered the protocol in PROSPERO (ID: CRD42019129310). Additional information about the methods is provided in the appendix (Additional file 1: Supplementary Methods).

### Literature search strategy

We retrieved articles from the PubMed, Embase, and Cochrane databases to identify studies that reported the association between irAE occurrence and the efficacy of ICIs in patients with cancer that were published from database inception to March 22, 2019. The key retrieval items included irAEs, PD-1, PD-L1, CTLA-4, efficacy, and cancer. No restriction for time was established, while language was confined to English. We also manually reviewed the citations of relevant reviews, editorials, and commentaries and included relevant studies to avoid omission. We performed an additional retrieval from the database inception to June 3, 2019, to identify recent published studies using the same procedure.

### Inclusion and exclusion criteria

The following inclusion criteria were adopted:

- Studies that enrolled patients diagnosed with cancer who had been treated with at least one of the following ICIs: nivolumab, pembrolizumab, atezolizumab, durvalumab, avelumab, or ipilimumab
- Studies that reported an association between irAE occurrence and ICI efficacy in patients with cancer, including hazard ratios (HRs) of OS and progression-free survival (PFS) in patients who experienced irAEs versus non-irAE patients
- Studies that reported available survival data for the extraction of HRs and 95% confidence intervals (CIs) or *p* values
- Studies that enrolled patients who had received prior treatment or current combination treatment were eligible (e.g., chemotherapy, radiotherapy, and vaccine therapy)
- Prospective or retrospective cohort studies, including on-trial and off-trial patients
- Studies published in peer-reviewed journals in English.

Studies not adhering to the inclusion criteria were excluded. Other exclusion criteria were as follows:

- Studies that reported adverse events that were not related to immune function
- Studies that reported only survival curves and *p* values, but not HRs, for the association between the occurrence of irAEs and the efficacy of ICIs
- For duplicate publications or overlapping study populations, we included only the most recent and complete report.

### Data collection and quality assessment

Two researchers (X.Z. and Z.Y.) independently extracted data from the included publications in accordance with a predefined procedure. The data extracted included the author, publication year, area in which the population was located, trial design, criteria for grading irAEs, statistical model, variables for adjustment, landmark analysis, cancer type, agent, follow-up time, sample size, irAE type, grade of irAE, median irAE onset time, and HRs and 95% CIs of OS and PFS for global irAEs, organ-specific irAEs, and grade-specific irAEs. If a study reported both multivariate and univariate HRs, the former was extracted to avoid confounding. If a study reported both HRs with or without a landmark analysis, the former was chosen to avoid time-dependent bias.

The two researchers (X.Z. and Z.Y.) also independently reviewed the included publications to evaluate their methodological quality with the Newcastle-Ottawa scale (NOS) criteria [38]. Every included study was awarded a score ranging from 0 (poor methodological quality) to 9 (optimal methodological quality) points regarding the selection, comparability and outcomes of study cohorts. Any discrepancies were resolved by reaching a consensus with a third author (H.Y. or N.L.).

### Data analyses

We utilized Stata 12.0 software (Stata Corporation, College Station, Texas, USA) and R gui software (version 3.4.4), with the forestplot_v.1.7.2 package for statistical analyses and plotting. The log HRs of irAEs versus non-irAEs and 95% CIs were adopted to aggregate the survival results. If a study reported only HRs and *p* values, but not 95% CIs, the conversion formula proposed by Altman et al. was utilized to calculate the 95% CIs [39]. If an HR of non-irAEs versus irAEs rather than the opposite comparison was reported, then an HR of irAEs versus non-irAEs was calculated by determining the reciprocal of original HR and corresponding CIs [40]. The *χ*^2^ test and *I*^2^ statistic were applied to estimate the between-study heterogeneity. Significant heterogeneity was indicated if *p <* 0.10 for the *χ*^2^ test or *I*^2^ > 50%, and a random effects model was applied to the pooled analysis [41]. Otherwise, we applied a fixed effects model [42]. For the sensitivity analysis, one study was sequentially omitted to judge the stability of the pooled results. Begg’s test and Egger’s test were utilized to identify publication bias [43, 44]. For both tests, significant publication bias was considered when *p <* 0.05. Moreover, the “trim and fill” method was adopted to identify and adjust for potential sources of publication bias [45]. This method estimated possibly missing studies and incorporated these hypothetical studies into the original analysis to calculate an adjusted effect. For all analyses, a two-sided *p <* 0.05 was considered representative of statistical significance.

We pooled the studies for organ-specific irAEs, low-grade irAEs (grades 1–2), and severe-grade irAEs (grade greater than or equal to 3) if at least two studies were identified. We also pooled HRs from all studies to identify the overall effect. For the overall pooled analysis, if a study reported both HRs of all-grade irAEs and grade-specific irAEs, the former was selected; if a study reported both HRs of global irAEs and organ-specific irAEs, the former was selected; and if a study reported HRs of multiple organ-specific irAEs, but not of global irAEs, the analysis with the largest sample size of the irAE cohort was selected.

We performed predefined subgroup analyses to investigate the effects of irAEs on the efficacy of ICIs among different settings and potential sources of heterogeneity. We considered subgroups, including cancer type, class of ICIs, combination therapy, sample size, model, landmark analysis, and approach for data extraction, to the overall cohort. In addition, we performed a subgroup analysis of the class of ICIs in the melanoma cohort. We only analyzed subgroups containing at least two studies.

## Results

### Literature search results

**Fig. 1:**
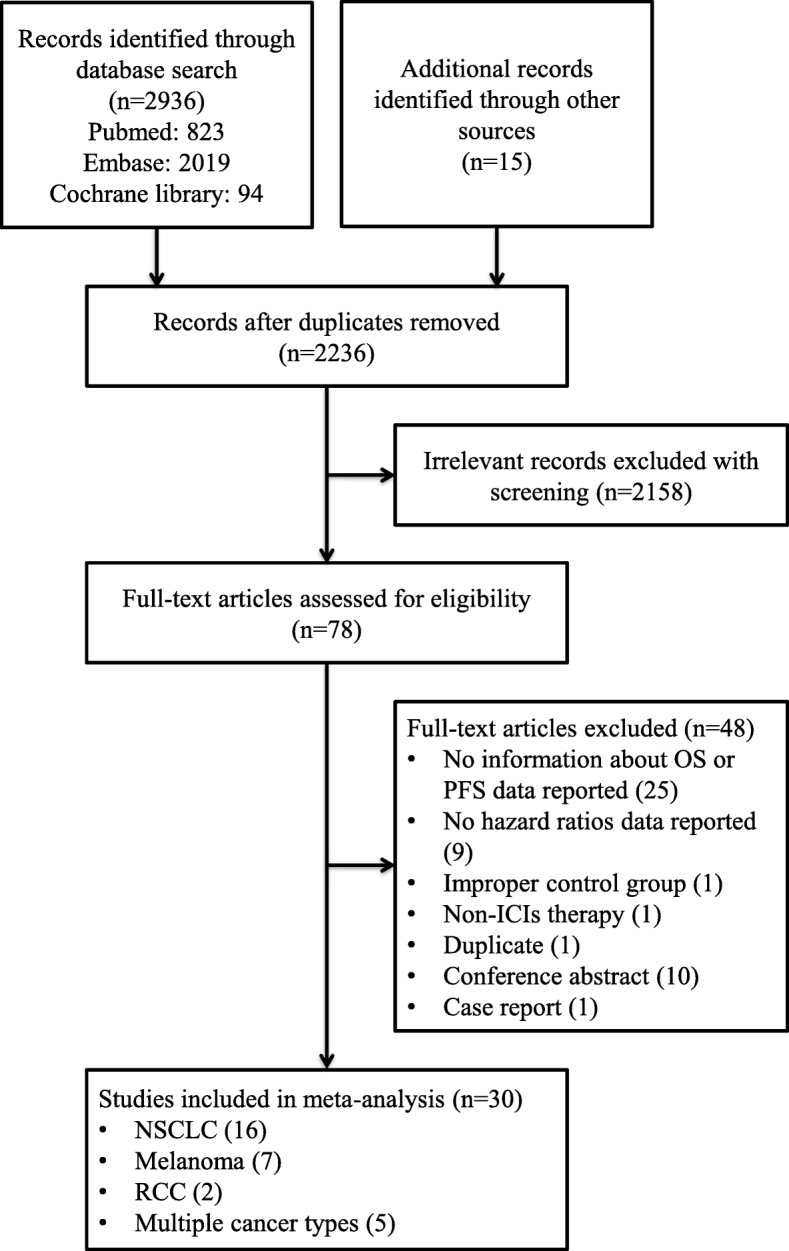
Flowchart of study selection process. Abbreviations: OS, overall survival; PFS, progression-free survival; NSCLC, non-small cell lung cancer; RCC, renal cell carcinoma

Our literature search retrieved 2236 studies, from which we collected 78 potentially eligible studies after screening the titles and abstracts. Finally, we selected 30 studies after a review of the full article [4–33]. The reasons for exclusion were as follows: Twenty-five studies did not report data on OS or PFS, 9 did not report data of HRs, 1 was a duplicate publication, 1 used an improper control group, 1 reported non-ICI therapy, 1 was a published conference abstract, and 1 was a case report. The detailed retrieval process is shown in Fig. 1.

### Characteristics of the identified studies

We retrieved information from 4971 individuals in this study. The characteristics of the 30 included studies are described in Table 1 and Additional file 1: Tables S1 and S2. These studies were performed in 10 countries. Sixteen studies analyzed patients with non-small cell lung cancer (NSCLC), 7 analyzed patients with melanoma, 2 analyzed patients with renal cell carcinoma (RCC), and 5 analyzed patients with multiple cancer types. Twenty-six studies adopted anti-PD-1 inhibitors, 3 adopted anti-CTLA-4 inhibitors, and 1 adopted anti-PD-1/PD-L1 inhibitors. The number of patients included in the survival analysis ranged from 18 to 613. Twenty studies reported extractable data on global irAEs, and 16 studies reported organ-specific irAEs. Twenty-six studies reported HRs of OS and 22 studies reported HRs of PFS. Six studies utilized a landmark analysis, whereas 24 studies did not. Seventeen studies adopted a multivariate model to control for confounding factors, and 13 studies adopted a univariate model. Two studies were had a prospective cohort design, and 28 studies employed a retrospective cohort design. All studies enrolled patients with stage III or higher cancer, except two studies that did not report this information. Four studies included on-trial patients, 22 studies included off-trial patients, and 4 studies included both on-trial and off-trial patients. The median irAE onset time ranged from 4.2 to 20 weeks. Twenty-six studies adopted ICIs as monotherapy, and 4 studies adopted ICIs as combination therapy (2 with a peptide vaccine, 1 with radiotherapy, and 1 with vemurafenib). All studies adopted the Common Terminology Criteria for Adverse Events (CTCAE) to grade irAEs, with the exception of 3 studies that did not report this information. The NOS scores allocated for the included studies ranged from 4 to 8 points.

**Table 1:** Main characteristics of the eligible studies

Study	Cancer type	Agents	Exposed group/ total, No.	irAE type	irAE grade	Hazard ratio (95% CI)	Landmark analysis	Model	Design
Sanlorenzo, 2015 [32]	Multiple	P	19/43^a^	Skin	1–3	PFS: 0.82 (0.17–4.06) PFS: 0.70 (0.05–9.50) PFS: 0.12 (0.02–0.74)	No	M	RC
			7/24^b^		1–3				
			9/16^c^		1				
Keller, 2016 [9]	Melanoma	N	67/143	Rash	1–3	OS: 0.423 (0.243–0.735)	12 weeks	M	RC
			50/143	Pneumonitis	1–2	OS: 0.371 (0.022–6.313)			
			19/143	Vitiligo	1–2	OS: 0.184 (0.036–0.940)			
			16/143	Hypothyroidism	1–2	OS: 0.360 (0.100–1.291)			
			9/143	Mucositis	1–2	OS: 0.087 (0.005–1.448)			
			3/143	Diarrhea/colitis	1–3	OS: 0.632 (0.348–1.149)			
			2/143	Hyperthyroidism	1–2	OS: 1.604 (0.420–6.118)			
			N.A./143	Myalgias	1–2	OS: 0.377 (0.022–6.477)			
Haratani, 2017 [10]	NSCLC	N	OS: 46/130 PFS: 44/105	Global	1–4	OS: 0.285 (0.102–0.675) PFS: 0.542 (0.295–0.971)	6 weeks	M	RC
			OS: 31/130 PFS: 31/105	Skin	1–4	OS: 0.209 (0.049–0.618) PFS: 0.476 (0.232–0.912)			
			OS: 6/130 PFS: 6/105	Endocrine	1–4	OS: 0.504 (0.027–2.629) PFS: 0.237 (0.037–0.842)			
Kim, 2017 [11]	NSCLC	N/P	19/58	Thyroid dysfunction	1–2	OS: 0.11 (0.01–0.92) PFS: 0.38 (0.17–0.85)	No	M	RC
Judd, 2017 [23]	Multiple	N/P	N.A./173	Global	1–2	OS: 0.480 (0.227–1.107) ^d^	No	M	RC
Osorio, 2017 [12]	NSCLC	P	10/48	Thyroid dysfunction	1–3	OS: 0.29 (0.09–0.94) PFS: 0.58 (0.27–1.21)	No	U	PC
Nakamura, 2017 [22]	Melanoma	N	9/35	Vitiligo	1–2	OS: 0.16 (0.03–0.79) PFS: 0.58 (0.27–1.21)	No	U	RC
Grangeon, 2018 [14]	NSCLC	N/P	124/270	Global	1–4	OS: 0.29 (0.18–0.46) PFS: 0.42 (0.32–0.57)	No	U	RC
			53/270	Thyroiditis	1–4	OS: 0.46 (0.25–0.86) PFS: 0.58 (0.39–0.85)			
			11/270	Colitis	1–4	OS: 0.24 (0.03–1.73) PFS: 0.73 (0.35–1.50)			
			8/270	Hepatitis	1–4	OS: 0.97 (0.30–3.08) PFS: 0.94 (0.45–2.08)			
			6/270	Pneumonitis	1–4	OS: 1.42 (0.45–1.54) PFS: 1.19 (0.52–2.7)			
Toi, 2018 [18]	NSCLC	N/P	66/137	Global	1–4	OS: 0.42 (0.24–0.71) PFS: 0.45 (0.30–0.68)	No	U	RC
Sato, 2018 [31]	NSCLC	N	11/18^e^	Global	1–4	PFS: 0.28 (0.04–1.46)	60 days	U	RC
Rogado, 2018 [25]	Multiple	N/P	40/106	Global	1–4	OS: 0.909 (0.625–1.429)^f^ PFS: 0.435 (0.278–0.714)^f^	No	M	RC
Ricciuti, 2018 [15]	NSCLC	N	85/195	Global	1–4	OS: 0.38 (0.26–0.56) PFS: 0.48 (0.34–0.67)	No	M	RC
			39/195	Endocrine	1–2				
						OS: 0.59 (0.40–0.89) PFS: 0.46 (0.24–0.89)			
			32/195	Hepatobiliary	1–4	OS: 0.94 (0.53–1.66) PFS: 0.72 (0.41–1.24)			
			21/195	Skin	1–4	OS: 0.80 (0.46–1.39) PFS: 0.57 (0.35–0.95)			
			17/195	Gastrointestinal	1–4	OS: 0.52 (0.30–0.90) PFS:0.50 (0.26–0.98)			
			16/195	Lung	1–4	PFS: 0.45 (0.28–0.72) OS: 0.56 (0.33–0.96)			
Ksienski, 2018 [24]	NSCLC	N/P	91/246	Global	1–2	OS: 0.85 (0.50–1.42)	6 weeks	M	RC
			25/180		≥3	OS: 2.29 (1.05–4.98)			
Faje, 2018 [8]	Melanoma	I	64/281	Hypophysitis	N.A.	OS: 0.53 (0.36–0.75)	No	U	RC
Indini, 2018 [4]	Melanoma	N/P	102/173	Global	1–5	OS: 0.39 (0.18–0.81) PFS: 0.47 (0.26–0.86)	No	M	RC
Lesueur, 2018 [26]	NSCLC	N	62/104	Global	1–4	OS: 0.640 (0.377–1.087) PFS: 0.660 (0.433–1.099)	No	M	RC
Owen, 2018 [5]	NSCLC	N/P/A	27/91	Global	1–4	OS: 0.364 (0.203–0.649)^f^	No	U	RC
Lisberg, 2018 [27]	NSCLC	P	28/97	Global	1–4	OS: 0.72 (0.49–1.05) PFS: 0.62 (0.40–0.96)	No	M	RC
Fujimoto, 2018 [30]	NSCLC	N	68/613	Global	≥3	PFS: 0.76 (0.55–1.01)	No	M	RC
			62/613	Pneumonitis	1–4	PFS: 0.71 (0.52–0.97)			
Okada, 2019 [6]	Melanoma	N	8/15	Global	1–2	OS: 0.01 (0.00011–0.88)	No	M	RC
Lei, 2019 [16]	Multiple	N/P	34/103	Thyroiditis	1–4	OS: 0.40 (0.19–0.85) PFS: 0.45 (0.27–0.76)	No	U	RC
Cortellini, 2019 [19]	NSCLC	N/P	224/524	Global	1–4	OS: 0.55 (0.41–0.72) PFS: 0.59 (0.47–0.76)	6 weeks	M	RC
			50/559		3–4	OS: 0.53 (0.41–0.69) PFS: 0.75 (0.51–1.11)	No	M	
			78/559	Endocrine	1–4	OS: 0.55 (0.37–0.83) PFS: 0.63 (0.45–0.89)	No	M	
			59/559	Skin	1–4	OS: 0.43 (0.27–0.70) PFS: 0.46 (0.31–0.69)	No	M	
			51/559	Gastrointestinal	1–4	OS: 0.61 (0.38–0.98) PFS: 0.68 (0.47–1.01)	No	OS: M PFS: U	
			23/559	Pneumonitis	1–4	OS: 1.32 (0.79–2.19) PFS: 1.20 (0.76–1.92)	No	U	
			10/559	Hepatic	1–4	OS: 1.09 (0.48–2.45) PFS: 1.47 (0.72–2.96)	No	U	
Ahn, 2019 [21]	NSCLC	N/P	OS: 55/133 PFS: 51/111	Global	1–4	OS: 0.484 (0.255–0.919) PFS: 0.434 (0.256–0.735)	6 weeks	M	RC
			OS: 26/133 PFS: 24/133	Skin	1–2	OS: 0.420 (0.162–1.087) PFS: 0.643 (0.350–1.180)			
			OS: 14/133 PFS: 14/111	Endocrine	1–4	OS: 0.255 (0.051–1.288) PFS: 0.368 (0.132–1.028)			
			OS: N.A./133 PFS: N.A./111	Pneumonitis	1–4	OS: 4.177 (1.420–11.942) PFS: 1.686 (0.618–4.597)			
Berner, 2019 [20]	NSCLC	N/P	48/83	Skin	N.A.	OS: 0.29 (0.12–0.71) PFS: 0.22 (0.09–0.39)	No	U	PC
Verzoni, 2019 [7]	RCC	N	77/389	Global	1–4	OS: 0.57 (0.35–0.93)	No	M	RC
Yamauchi, 2019 [13]	Multiple	N	OS: 67/191 PFS: 61/175	Thyroid	N.A.	OS: 0.61 (0.39–0.93) PFS: 0.66 (0.46–0.95)	No	U	RC
Bjørnhart, 2019 [28]	NSCLC	N/P	25/112	Global	3–4	OS: 0.47 (0.21–1.05) PFS: 0.71 (0.39–1.27)	No	U	RC
Ishihara, 2019 [17]	RCC	N	23/47	Global	1–4	PFS: 0.25 (0.11–0.56)	No	M	RC
Moel, 2019 [33]	Melanoma	I	81/133 ^e^	Global	1–4	OS: 1.12 (0.7–1.79)	No	U	RC
Lang, 2019 [29]	Melanoma	I	29/100	Diarrhea	1–3	OS: 1.32 (0.71–2.44) PFS: 1.40 (0.88–2.22)	No	U	RC
			7/100	Diarrhea	3	OS: 2.15 (0.76–6.07) PFS: 1.96 (0.89–4.32)			

Abbreviations: *irAE* immune-related adverse event, *NSCLC* non-small-cell lung carcinoma, *RCC* renal cell carcinoma, *Multiple* multiple cancer types, *RC* retrospective cohort, *PC* prospective cohort, *N* nivolumab, *P* pembrolizumab, *A* atezolizumab, *I* ipilimumab, *N.A.* not available, *OS* overall survival, *PFS* progression-free survival, *M* multivariate, *U* univariate

^a^The patients group receiving a dose of 10 mg/kg every 3 weeks

^b^The patients group receiving a dose of 10 mg/kg every 2 weeks

^c^The patients group receiving a dose of 2 mg/kg every 3 weeks

^d^The 95% CI was calculated according to the HR and *p* value

^e^The sample size was estimated from the manuscript

^f^The HR and 95% CI was calculated through taking reciprocal

### Primary outcome: OS

**Fig. 2:**
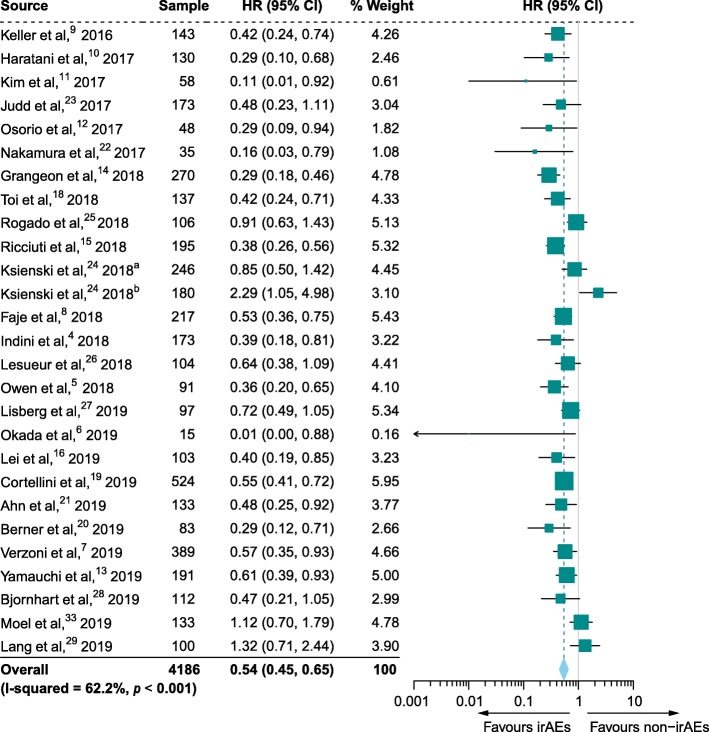
Forest plot (random effects model) of the association between immune-related adverse event development and overall survival. The sizes of the squares indicate the weight of the study. Abbreviations: HR, hazard ratio; irAEs, immune-related adverse events; non-irAEs, non-immune-related adverse events. ^a^Results for grade 1–2 immune-related adverse events (irAEs). ^b^Results for grade 3–4 irAEs

**Fig. 3:**
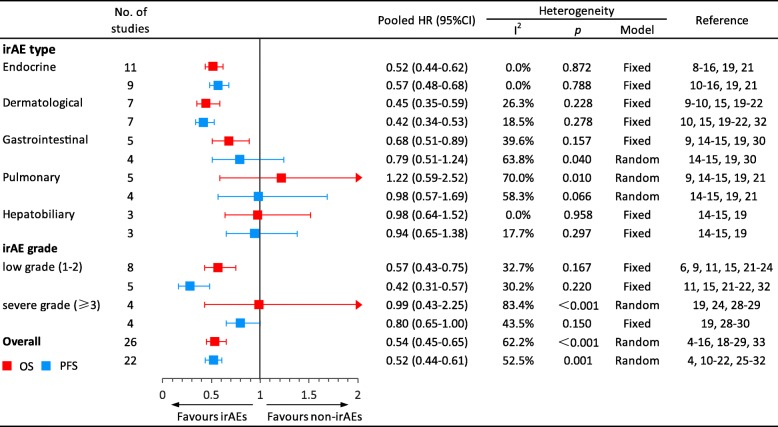
Meta-analyses of the association between immune-related adverse event development and outcome. Abbreviations: HR, hazard ratio; irAEs, immune-related adverse events; non-irAEs, non-immune-related adverse events. Low grade indicates grades 1–2; severe grade indicates a grade greater than or equal to 3. ^a^The HR was directly presented without pooling because only one study was available

Twenty-six studies comprising 4186 patients that reported HRs of OS were ultimately included in the pooled analysis. The irAE occurrence was significantly associated with improved OS in patients undergoing ICI therapy (HR, 0.54; 95% CI, 0.45–0.65; *p <* 0.001) (Figs. 2 and 3). However, significant heterogeneity was detected (*I*^2^ = 62.2%, *p <* 0.001).

We further separately pooled the HRs of OS according to the different types of irAEs. The occurrences of endocrine, dermatological, and gastrointestinal irAEs were significantly associated with a favorable OS in patients treated with ICIs (endocrine irAEs: HR, 0.52, 95% CI, 0.44–0.62, *p <* 0.001; dermatological irAEs: HR, 0.45, 95% CI, 0.35–0.59, *p <* 0.001; gastrointestinal irAEs: HR, 0.68, 95% CI, 0.51–0.89, *p* = 0.005). Nevertheless, the occurrences of pulmonary and hepatobiliary irAEs were not significantly associated with OS (pulmonary irAEs: HR, 1.22, 95% CI, 0.59–2.52, *p* = 0.584; hepatobiliary irAEs: HR, 0.98, 95% CI, 0.64–1.52, *p* = 0.944), with significant heterogeneity observed for pulmonary irAEs (*I*^2^ = 70.0%, *p <* 0.001), but not other irAE types. The occurrence of musculoskeletal irAEs also did not show a significant association with OS (HR, 0.38, 95% CI, 0.02–6.48, *p* = 0.502), although only one study was included (Fig. 3).

The HRs of OS for patients presenting with low and severe irAE grades were also analyzed. The occurrence of low-grade irAEs was significantly associated with a favorable OS in patients receiving ICIs (HR, 0.57, 95% CI, 0.43–0.75, *p <* 0.001), whereas the occurrence of severe-grade irAEs did not display a significant association with OS (HR, 0.99, 95% CI, 0.43–2.25, *p* = 0.976). Significant heterogeneity was observed in severe-grade irAEs (*I*^2^ = 83.4%, *p <* 0.001), but not in low-grade irAEs (Fig. 3).

**Fig. 4:**
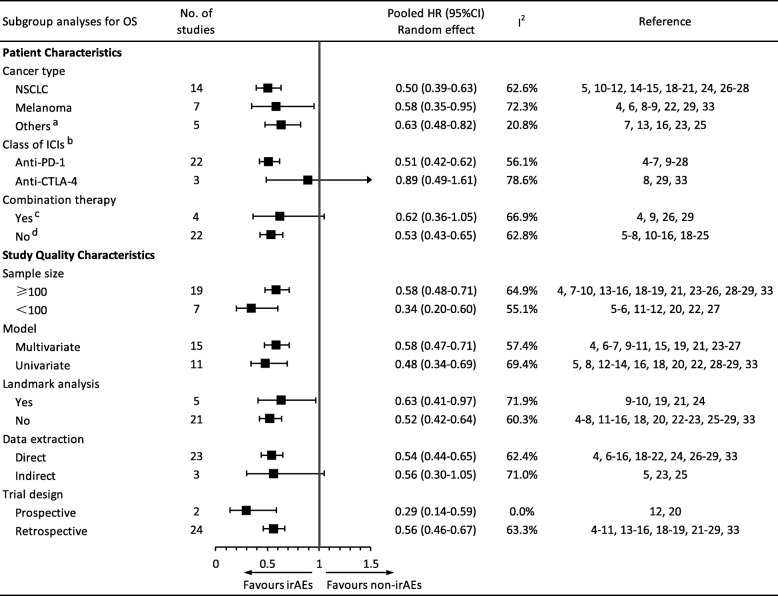
Subgroup analyses of the association between immune-related adverse event development and overall survival. Abbreviations: OS, overall survival; HR, hazard ratio; NSCLC, non-small cell lung cancer; ICIs, immune checkpoint inhibitors; anti-PD-1, anti-programmed cell death-1; anti-CTLA-4, anti-cytotoxic T-lymphocyte antigen-4. ^a^This group included four multiple cancer types and 1 renal cell carcinoma. ^b^The study reported by Owen et al. was not included in subgroup analysis regarding class of ICIs because it is the only one study investigating anti-PD-1/anti-PD-L1 drug. ^c^Yes indicates studies that combined ICIs with other therapy, including peptide vaccine (*n* = 2), radiotherapy (*n* = 1) and Vemurafenib (*n* = 1). ^d^No indicates studies that adopted ICIs as monotherapy

Predefined subgroup analyses were performed according to a series of study quality characteristics and patient characteristics (Fig. 4). The subgroups stratified by study quality characteristics did not change the results, although subgroups stratified by patient characteristics yielded inconsistent results. For example, patients with cancer who were treated with ICIs as a monotherapy, but not combination therapy, experienced a significant OS benefit when irAEs occurred (monotherapy: HR, 0.53, 95% CI, 0.43–0.65, *p <* 0.001; combination therapy: HR, 0.62, 95% CI, 0.36–1.05, *p* = 0.073). In addition, the occurrence of irAEs predicted a favorable OS in patients with cancer receiving PD-1 inhibitors (HR, 0.51, 95% CI, 0.40–0.64, *p <* 0.001), but not CTLA-4 inhibitors (HR, 0.89, 95% CI, 0.49–1.61, *p* = 0.706). Additionally, significant predictive effects of irAEs on a favorable OS were observed in all subgroups stratified by cancer types, although in patients with melanoma, the effect displayed borderline to statistical threshold value (NSCLC: HR, 0.50, 95% CI, 0.39–0.63, *p <* 0.001; melanoma: HR, 0.58, 95% CI, 0.35–0.95, *p* = 0.032; others: HR, 0.63, 95% CI, 0.48–0.82, *p* = 0.001). Additional subgroup analyses restricting the melanoma cohort to homogeneous class of ICIs revealed that the prediction was significant for patients with melanoma undergoing treatment with PD-1 inhibitors (HR, 0.35, 95% CI, 0.21–0.61, *p <* 0.001), but not CTLA-4 inhibitors (HR, 0.89, 95% CI, 0.49–1.61, *p* = 0.706) (Additional file 1: Figure S1).

### Secondary outcome: PFS

Twenty-two studies comprising 3297 patients that reported HRs of PFS were included. Similar to OS, the occurrence of irAEs were significantly associated with a favorable PFS in patients receiving ICIs (HR, 0.52, 95% CI, 0.44–0.61, *p <* 0.001), but was accompanied by significant heterogeneity (*I*^2^ = 52.5%, *p* = 0.001) (Fig. 3 and Additional file 1: Figure S2).

In the analysis of different types of irAEs, the occurrence of endocrine and dermatological irAEs was significantly associated with a favorable PFS in patients treated with ICIs (endocrine irAEs: HR, 0.57, 95% CI, 0.48–0.68, *p <* 0.001; dermatological irAEs: HR, 0.42, 95% CI, 0.34–0.53, *p <* 0.001). However, gastrointestinal, pulmonary, and hepatobiliary irAEs were not significantly associated with a favorable PFS (gastrointestinal irAEs: HR, 0.79, 95% CI, 0.51–1.24, *p* = 0.303, pulmonary irAEs: HR, 0.98, 95% CI, 0.57–1.69, *p* = 0.949; hepatobiliary irAEs: HR, 0.94, 95% CI, 0.65–1.38, *p* = 0.762) (Fig. 3). Significant heterogeneity was detected in gastrointestinal (*I*^2^ = 63.8%, *p* = 0.040) and pulmonary irAEs (*I*^2^ = 58.3%, *p* = 0.066), but not in the other irAE types.

Regarding the grades of irAEs, the pooled analysis showed that both low-grade irAEs and severe-grade irAEs were significantly associated with a favorable PFS, although the significance of severe-grade irAEs was marginal (low grade: HR, 0.42, 95% CI, 0.31–0.57, *p <* 0.001; severe grade: HR, 0.80, 95% CI, 0.65–1.00, *p* = 0.045) (Fig. 3), with no significant heterogeneity observed.

The results of subgroup analyses were similar to OS (Additional file 1: Figure S3). The association of irAE occurrences with a reduced risk of progression in patients with cancer receiving ICIs was significant in each subgroup stratified by study quality characteristics. Nonetheless, the predictive effect of irAEs on a favorable PFS was not consistently significant in subgroups stratified by patient characteristics.

### Sensitivity analysis and publication bias

In the sensitivity analysis, the pooled results for OS and PFS both remained significant, regardless of which study was deleted, indicating that the significant association between irAE occurrence and ICI efficacy in patients with cancer was robust (Additional file 1: Figure S4). Regarding the overall analysis, the Begg funnel plot for OS displayed evident asymmetry (*p* = 0.033), indicating that publication bias should be considered, although Egger’s test showed no evidence of publication bias (*p* = 0.122) (Additional file 1: Figure S5A). Next, we used the trim and fill method to assess the effect of publication bias on the pooled results. However, no study was trimmed or filled in the output results, leaving the pooled result of OS unchanged, which supported the stability of results (Additional file 1: Figure S5A and Additional file 2). Because publication bias is generally caused by small-sized studies, restricting the pooled analysis to large-sized studies (≥100) might provide clues for the origin of publication bias. Indeed, the pooled HR of OS in the large-sized studies was comparable to the overall effect (0.58 vs. 0.54) (Fig. 4). Notably, neither the Begg funnel plot (*p* = 0.721) nor Egger’s test (*p* = 0.872) revealed publication bias for OS in large-sized studies, which further confirmed the stability of the OS results (Additional file 1: Figure S6). Regarding PFS, the Begg funnel plot displayed no obvious asymmetry (*p* = 0.180), indicating that no evident publication bias was detected, and Egger’s test confirmed this finding (*p* = 0.134) (Additional file 1: Figure S7).

## Discussion

### Principal findings and implications

Currently, a decision regarding whether the occurrence of irAEs is associated with ICI therapy remains controversial. To our knowledge, our study represents the largest and most comprehensive analysis of the association between irAEs and ICI efficacy performed to date. The conclusions listed below were drawn based on our results.

- Generally, patients with cancer who developed irAEs experienced an increased OS and PFS compared with patients who did not develop irAEs.
- Regarding the irAE types, the survival benefit for patients who developed irAEs was observed in patients presenting endocrinal and dermatological abnormalities, but not in patients presenting a gastrointestinal, pulmonary, hepatobiliary, or musculoskeletal abnormality.
- The occurrence of low-grade irAEs, but not severe-grade irAEs, was associated with better ICI efficacy in patients with cancer.
- The occurrence of irAEs was significantly associated with a favorable efficacy of PD-1 inhibitors, but not CTLA-4 inhibitors.

The mechanisms underlying the association between irAEs and survival benefits have not been completely elucidated. Antigen mimicry theory has been one of the most promising hypotheses. Preclinical data identified multiple epitopes that were shared in both melanoma and normal melanocytes [46, 47]. The release of shared antigens by ICI therapy might result in the priming of a secondary immune response to host antigens, which was supported by the finding that T cell clones infiltrating irAE lesions and tumors were significantly overlapped among ICI-treated patients with melanoma and NSCLC [48]. Hence, the development of irAEs indicates a robust immune reaction towards both the tumor and healthy tissue, thereby predicting better treatment responses. Moreover, in addition to the antigen mimicry theory, the dysregulation of humoral immunity has been proposed as a possible explanation for the association. The PD-1 signaling pathway modulates B cell activation in both a T cell-dependent and T cell-independent manner [49, 50]. According to the clinical evidence, thyroid dysfunction during ICI treatment is characterized by the production of anti-thyroid antibodies [12], suggesting that the presence of autoantibodies may account for the irAE-prognosis relationship.

Notably, in addition to overall irAEs, the favorable results remained significant for endocrine irAEs and dermatological irAEs, but not for gastrointestinal irAEs, pulmonary irAEs, hepatobiliary irAEs, and musculoskeletal irAEs. As the incidence of musculoskeletal irAEs is low [51], statistical significance may not have been reached due to the insufficient number of samples. Gastrointestinal irAEs are more frequently observed in response to anti-CTLA-4 treatment [52], and thus, the insignificance of pooled results for PFS might be explained by heterogeneity. However, the variation among other organ-specific irAEs is likely attributable to the clinical importance of different systems. The respiratory and hepatobiliary systems are the most commonly affected organs in patients who experienced fatal irAEs and received anti-PD-1/PD-L1 treatment [53], increasing the risk of mortality for the patient and leading to a poorer outcome due to the side effects of ICIs. Therefore, the proper management of irAEs is very important to maximize the benefits of ICIs.

The prognostic value of irAEs also differed in patients with heterogeneous irAE grades, i.e., the predictive effect of irAEs was significant on low-grade irAEs but not severe-grade irAEs. Severe irAEs are potentially life-threatening and require systemic immunosuppressive treatment, which may counteract the effect of ICIs. Glucocorticoids extensively modify cytokine signaling and inhibit the IL-2 and INF-γ pathways [54–56], which are reactivated to create the inflammatory tumor microenvironment during ICI therapy. Therefore, exposure to large amounts of immunosuppressive reagents during high-grade irAEs would be expected to alter the antitumor effect.

Regarding the subgroup analyses, the distinct outcomes observed for the anti-PD-1 and anti-CTLA-4 subgroups indicated a possibly different mechanism of irAEs, as CTLA-4 blockade activates T cells at an earlier stage of their development and might thus directly disrupt central tolerance without affecting the tumor immune response. Meanwhile, irAEs induced by PD-1 inhibitors predict a better clinical response by patients with cancer, but the association between irAEs and survival in patients undergoing anti-CTLA-4 therapy remains controversial [8, 29, 57–60], demanding larger size of studies in the future. The ability of irAEs to predict a favorable OS and PFS was consistently significant in patients with NSCLC and other cancers (including RCC and multiple cancer types), but not in patients with melanoma. However, we prefer to attribute this inconsistency in patients with melanoma to the heterogeneity caused by the large proportion of studies investigating CTLA-4 inhibitors (OS: 3 in 7; PFS: 1 in 3), as the additional subgroup analysis revealed that restricting the melanoma cohort to treatment with PD-1 inhibitors yielded significant association between irAE development and a favorable OS.

### Strengths and comparison with other studies

A systematic review performed by Ouwerkerk et al. summarized studies investigating the association between irAEs and ICI efficacy in patients with melanoma [36]. However, the report by Ouwerkerk et al. did not pool the results through a meta-analysis and thus did not provide exact statistical information about the critical question of whether the occurrence of irAEs was associated with ICI efficacy. Additionally, the research scope of their study was restricted to patients with melanoma. In the present systematic review and meta-analysis, we performed a comprehensive pan-cancer meta-analysis. Therefore, an accurate magnitude of the predictive effect of irAEs on ICI efficacy was obtained. Additionally, we separately pooled the predictive effects of different irAE types and irAE grades to investigate their specific roles in homogeneous settings.

The relevance of our findings is strengthened by their consistency across all analyzed subsets stratified by the study quality characteristics, except for data extraction. Nevertheless, some of the included studies utilized a univariate model or were small in size, and a landmark analysis was not performed in every study we selected. However, as described above, the subgroup HR value remained significant, regardless of the adjustment for the model, sample size, or landmark analysis, indicating that the biases derived from these low-quality studies are unlikely to change our results.

### Limitations

Several limitations of our study should be noted. First, the Begg funnel plot and Begg’s test identified evident publication bias in the pooled results for OS, indicating that the results of the pooled analysis of OS might be exaggerated. According to the exclusion criteria, we excluded several major studies from the current meta-analysis because they reported only survival curves but not HR values. Two studies presented no significant difference in survival outcome based on irAEs [34, 35], but one study reported a significant difference in survival according to irAEs with landmark analysis [61]. However, Egger’s test and the trim and fill method detected no evidence of publication bias. Compared with the overall analysis of OS, the pooled analysis of OS in large-sized studies was comparable and showed no publication bias, indicating that the bias attributed to small-sized studies was moderate. In addition, the PFS analysis showed a similar significant overall effect to the OS analysis and exhibited no publication bias. Taken together, we believe that our study provides meaningful evidence specifying the association of irAE development with survival benefits in patients with cancer who were treated with ICIs. Second, significant heterogeneity was observed in the OS analysis, which might be attributed to variations in irAE types, irAE grade, class of ICIs, etc. In an attempt to reduce the impact of heterogeneity, we performed specific analyses of each type and grade of irAEs. We also conducted subgroup analyses of patient characteristics and study quality characteristics. Third, our study included limited types of malignancy that were mostly weighted towards NSCLC, melanoma, and RCC, restricting the broad application of our findings. Additional analyses of broader types of cancer are necessary to confirm our conclusions. Fourth, only two publications included in our study employed a prospective design, raising the concerns regarding the quality of evidence analyzed in our study. Hence, further large-scale prospective cohort studies are warranted.

## Conclusions

In this systematic review and meta-analysis, the occurrence of irAEs was associated with better ICI efficacy in patients with cancer, particularly endocrine, dermatological, and low-grade irAEs. Further large-scale prospective studies are warranted to confirm our discoveries.

## Supplementary information

Additional file 1. Contains additional information about the methods, literature search and data analyses. **Table S1.** Additional characteristics of the eligible studies. **Table S2.** The Newcastle-Ottawa Scale (NOS) quality assessment of the enrolled studies. **Figure S1.** Subgroup analysis stratified by class of immune checkpoint inhibitors in the melanoma cohort. **Figure S2.** Forest plot (fixed effects model) of the association between immune-related adverse event development and progression-free survival. **Figure S3.** Subgroup analyses of the association between immune-related adverse event development and progression-free survival. **Figure S4.** Sensitivity analysis of the impact of each individual study on the pooled effect. A) Overall survival; B) Progression-free survival. **Figure S5.** Funnel plots of the overall survival results. (A) Without trim and fill; (B) With trim and fill. **Figure S6.** Funnel plots of the overall survival results in large sample size studies. **Figure S7.** Funnel plots of the progression-free survival results.

Additional file 2. Log file of trim and fill method in Figure S5.

## Abbreviations

CIs: Confidence intervals
CTCAE: Common Terminology Criteria for Adverse Events
CTLA-4: Cytotoxic T-lymphocyte antigen-4
HRs: Hazard ratios
ICIs: Immune checkpoint inhibitors
irAEs: Immune-related adverse events
NOS: Newcastle-Ottawa scale
NSCLC: Non-small cell lung cancer
OS: Overall survival
PD-1: Programmed cell death-1
PD-L1: Programmed cell death ligand 1
PFS: Progression-free survival
RCC: Renal cell carcinoma
