# Supplementary material for: Are immune-related adverse events associated with the efficacy of immune checkpoint inhibitors in patients with cancer? A systematic review and meta-analysis
Source: BMC Med. 2020 Apr 20;18:87. doi: 10.1186/s12916-020-01549-2 (PMC7169020; doi:10.1186/s12916-020-01549-2)
Supplement: Supplementary file 1 — Contains additional information about the methods, literature search and data analyses. Table S1. Additional characteristics of the eligible studies. Table S2. The Newcastle-Ottawa Scale (NOS) quality assessment of the enrolled studies. Figure S1. Subgroup analysis stratified by class of immune checkpoint inhibitors in the melanoma cohort. Figure S2. Forest plot (fixed effects model) of the association between immune-related adverse event development and progression-free survival. Figure S3. Subgroup analyses of the association between immune-related adverse event development and progression-free survival. Figure S4. Sensitivity analysis of the impact of each individual study on the pooled effect. A) Overall survival; B) Progression-free survival. Figure S5. Funnel plots of the overall survival results. (A) Without trim and fill; (B) With trim and fill. Figure S6. Funnel plots of the overall survival results in large sample size studies. Figure S7. Funnel plots of the progression-free survival results. [file 12916_2020_1549_MOESM1_ESM.pdf]

Additional file 1

Supplementary Methods. Literature search and data analyses.

Table S1. Additional characteristics of the eligible studies.

Table S2. The Newcastle-Ottawa Scale (NOS) quality assessment of the enrolled studies.

Figure S1. Subgroup analysis stratified by class of immune checkpoint inhibitors in the melanoma cohort.

Figure S2. Forest plot (fixed effects model) of the correlation between immune-related adverse event development and progression-free survival.

Figure S3. Subgroup analyses of the correlation between immune-related adverse event development and progression-free survival.

Figure S4. Sensitivity analysis of the impact of each individual study on the pooled effect. A) Overall survival; B) Progression-free survival.

Figure S5. Funnel plots of the overall survival results. (A) Without trim and fill; (B) With trim and fill.

Figure S6. Funnel plots of the overall survival results in large sample size studies.

Figure S7. Funnel plots of the progression-free survival results.

Supplementary Methods. Literature search and data analyses

Literature search

A systematic literature search in the PubMed, EMBASE and Cochrane databases was conducted on March 22, 2019. The complete search strategy was ("irAEs" OR "immune-related adverse events" OR "treatment-related adverse events" OR "treatment-related AEs" OR "select adverse events" OR "select AEs" OR "select treatment-related adverse events" OR "select treatment-related AEs" OR "immune-mediated adverse events" OR "immune-mediated AEs") AND ("efficacy" OR "benefit" OR "response" OR "outcome" OR "prognosis") AND ("anti-PD-1" OR "PD-1" OR "anti-PD-L1" OR "PD-L1" OR "CTLA-4" OR "anti-CTLA-4" OR "nivolumab" OR "pembrolizumab" OR "atezolizumab" OR "durvalumab" OR "avelumab" OR "ipilimumab" OR "immune checkpoint inhibitor") AND ("cancer" OR "tumor" OR "tumour" OR "neoplasm" OR "carcinoma"). An additional retrieval was conducted from inception to June 3, 2019 to identify recent published studies.

Data analyses

According to the predefined protocol, subgroup analyses of OS were conducted for cancer type, class of ICIs, combination therapy, sample size, model, landmark analysis and approach of data extraction. Unlike OS, a subgroup analysis of PFS was not conducted for the class of ICIs because the ICIs investigated in 17 included studies were all classified as anti-PD-1 antibodies. Similarly, the approach of data extraction was also not considered for subgroup analyses because all 17 included studies, except one study, extracted data directly.

Supplementary Table 1: Additional characteristics of the eligible studies.

Study	irAE type	Country	Cancer stage	Patient data source	Median irAE onset time (weeks)	Combination therapy	Grading criteria	Adjusted variables
Sanlorenzo,[32] 2015	Skin	USA	Advanced	On-trial	NR	No	CTCAE 4.0	Treatment cycles
Keller,[9] 2016	Rash	USA	III-IV	On-trial	5.6	Peptide vaccine[a]	CTCAE 4.0	Age, sex
	Pneumonitis				10.9			
	Vitiligo				5.4			
	Hypothyroidism				10.7			
	Mucositis				9.7			
	Diarrhea/colitis				4.2			
	Hyperthyroidism				9.1			
	Myalgias				NR			
Haratani,[10] 2017	Global	Japan	IIIB-IV	Off-trial	4.1	No	CTCAE 4.0	Sex, age, treatment lines, smoking status, mutational status, brain metastasis
	Skin				5.7			
	Endocrine				4.6			
Kim,[11] 2017	Thyroid dysfunction	Korea	IV	Mixed	5.7	No	CTCAE 4.0	Age, sex, current or former smoking status, pathological subtypes, stage, medication types
Judd,[23] 2017	Global	USA	NR	Mixed	NR	No	CTCAE	Trial participation status, cancer type
Osorio,[12] 2017	Thyroid dysfunction	USA	IV	On-trial	6.0	No	CTCAE 4.0	No
Nakamura,[22] 2017	Vitiligo	Japan	III-IV	Mixed	20.0	No	CTCAE 4.0	No
Grangeon,[14] 2018	Global	France	Advanced	Off-trial	NR	No	CTCAE 4.0	No
	Thyroiditis							
	Colitis							
	Hepatitis							
	Pneumonitis							
Toi,[18] 2018	Global	Japan	Advanced	Off-trial	4.7	No	CTCAE 4.0	No
Sato,[31] 2018	Global	Japan	IIIB-IV	Off-trial	7.1	No	CTCAE 4.0	No
Rogado,[25] 2018	Global	Spain	NR	Off-trial	NR	No	CTCAE 4.0	Age, sex, histology, ECOG PS, smoking habit, treatment lines, type of anti-PD-1 antibody
Ricciuti,[15] 2018	Global	Italy	Advanced or recurrent	Off-trial	NR	No	CTCAE 4.0	Age, ECOG PS, brain metastasis
	Endocrine				7.8			
	Hepatobiliary				21			
	Skin				9.8			
	Gastrointestinal				9.0			
	Lung				16.2			
Ksienski,[24] 2018	Global	Canada	IV	Off-trial	NR	No	CTCAE 4.0	Age, sex, smoking status, CCI score, ECOG PS, liver

Supplementary Table 1: continued.

Study	irAE type	Country	Cancer stage	Patient data source	Median irAE onset time (weeks)	Combination therapy	Grading criteria	Adjusted variables
								metastases, brain metastases, line of therapy for PD-1 antibody
Faje,[8] 2018	Hypophysitis	USA	IIIA-IV	Off-trial	9.8	No	NR	Age, glucocorticoid dose, sex, serum LDL, tumor status, ECOG PS
Indini,[4] 2018	Global	Italy	IV	Off-trial	NR	Peptide vaccine[a]	CTCAE 4.0	Age, site of primary melonoma, metastatic sites, neutrophil to lymphocyte ratio, Lymphocyte to monocyte ratio, Lymphocyte ratio, LDH level
Lesueur,[26] 2018	Global	France	IV	Off-trial	NR	Radiotherapy[b]	CTCAE 4.0	ECOG PS
Owen,[5] 2018	Global	USA	Advanced	Mixed	12.0	No	CTCAE 4.0	No
Lisberg,[27] 2018	Global	USA	Locally advanced or metastatic	On-trial	NR	No	CTCAE 4.0	Age, sex, treatment lines, PD-L1 proportion score, EGFR status, smoking status, histology
Fujimoto,[30] 2018	Global<br>Pneumonitis	Japan	IIIB-IV	Off-trial	8.0	No	CTCAE 4.0	Age, sex, smoking status, ECOG PS, EGFR/ALK status, stage, treatment line, histology
Okada,[6] 2019	Global	Japan	III-IV	Off-trial	NR	No	CTCAE 4.0	Neutrophil count, Age
Lei,[16] 2019	Thyroiditis	USA	III-IV	Off-trial	NR	No	CTCAE 4.0	No
Cortellini,[19] 2019	Global<br>Endocrine<br>Skin<br>Gastrointestinal<br>Pneumonitis<br>Hepatic	Italy	Advanced	Off-trial	NR	No	CTCAE 4.0	OS: ECOG-PS, sex<br>PFS: ECOG-PS, treatment lines, metastatic sites
Ahn,[21] 2019	Global<br>Skin<br>Endocrine<br>Pneumonitis	Korea	Advanced	Off-trial	NR	No	CTCAE 4.0	Age, sex, ECOG PS, smoking status, PD-L1 expression, liver metastasis, brain metastasis, treatment lines, EGFR/ALK status
Berner,[20] 2019	Skin	Switzerland	Advanced	Off-trial	NR	No	NR	No
Verzoni,[7] 2019	Global	Italy	Metastatic	Off-trial	6w	No	CTCAE 4.0	Age, number of nivolumab doses
Yamauchi,[13] 2019	Thyroid	Japan	Advanced	Off-trial	NR	No	NR	No
Bjørnhart,[28] 2019	Global	Denmark	IIIA-IVB	Off-trial	NR	No	CTCAE 4.0	No

Supplementary Table 1: continued.

Study	irAE type	Country	Cancer stage	Patient data source	Median irAE onset time (weeks)	Combination therapy	Grading criteria	Adjusted variables
Ishihara,[17] 2019	Global	Japan	Metastatic	Off-trial	5.5 (1.8-15.6)	No	CTCAE 4.0	Sex, histopathology, MSKCC risk
Moel,[33] 2019	Global	Netherland	IIIC-IV	Off-trial	NR	No	CTCAE 4.0	No
Lang,[29] 2019	Diarrhea	Germany	IV	Off-trial	5.2 (0.3-13.1)	Vemurafenib[a]	CTCAE 4.0	No

Abbreviations: irAE, immune-related adverse event; USA, the United States of America; NR, not reported; CTCAE, The Common Terminology Criteria for Adverse Events; PD-1, programmed cell death 1; LDL, low density lipoprotein; ECOG PS, Eastern Cooperative Oncology Group Performance Status; CCI, Charlson comorbidity index; PD-L1, programmed cell death ligand 1; EGFR, epidermal growth factor receptor; ALK, anaplastic lymphoma kinase; OS, overall survival; PFS, progression-free survival; MSKCC, Memorial Sloan-Kettering Cancer Center.

[a] Combination therapy was adopted in part of the included cohort.

[b] Combination therapy was adopted in all of the included cohort.

Table S2: The Newcastle-Ottawa scale (NOS) quality assessment of the enrolled studies.

**Study ID**	**SELECTION**				**COMPARABILITY**	**OUTCOME**			**Total**[a]
	**Representative-ness of the exposed cohort**	**Selection of the non-exposed cohort**	**Ascertainment of exposure**	**Demonstration that outcome of interest was not present at start of study**[b]	**Comparability of cohorts on the basis of the design or analysis**[c]	**Assessment of outcome**	**Was follow-up long enough for outcomes to occur**[d]	**Adequacy of follow up of cohorts**[e]	
Sanlorenzo,[32] 2015	truly*	same institute*	record*	no	*	record*	no	yes*	6
Keller,[9] 2016	somewhat*	same institute*	record*	no	**	record*	no	yes*	7
Haratani,[10] 2017	somewhat*	same institute*	record*	no	**	record*	no	not clear	6
Kim,[11] 2017	somewhat*	same institute*	record*	no	*	record*	no	not clear	5
Judd,[23] 2017	truly*	same institute*	record*	no	*	record*	yes*	not clear	6
Osorio,[12] 2017	somewhat*	same institute*	record*	yes*	-	record*	yes*	not clear	6
Nakamura, [22] 2017	somewhat*	same institute*	record*	no	-	record*	no	not clear	4
Grangeon,[14] 2018	somewhat*	same institute*	record*	no	-	record*	no	not clear	4
Toi,[18] 2018	somewhat*	same institute*	record*	no	-	record*	no	not clear	4
Sato,[31] 2018	somewhat*	same institute*	record*	no	*	record*	no	not clear	5
Rogado,[25] 2018	truly *	same institute*	record*	no	*	record*	yes*	yes*	7
Ricciuti,[15] 2018	somewhat*	same institute*	record*	no	*	record*	yes*	not clear	6
Ksienski,[24] 2018	somewhat*	same institute*	record*	no	**	record*	yes*	not clear	7
Faje,[8] 2018	somewhat*	not clear	record*	no	*	record*	yes*	not clear	5
Indini,[4] 2018	somewhat*	same institute*	record*	no	*	record*	no	yes*	6
Lesueur,[26] 2018	somewhat*	same institute*	record*	no	*	record*	not clear	not clear	5
Owen,[5] 2018	somewhat*	same institute*	record*	no	-	record*	yes*	not clear	5
Lisberg,[27] 2018	somewhat*	same institute*	record*	no	*	record*	yes*	yes*	7
Fujimoto,[30] 2018	somewhat*	same institute*	record*	no	*	record*	not clear	not clear	5
Okada,[6] 2019	somewhat*	same institute*	record*	no	*	record*	no	not clear	5
Lei,[16] 2019	truly*	same institute*	record*	no	-	record*	no	not clear	4
Cortellini,[19] 2019	somewhat*	same institute *	record*	no	**	record*	yes*	not clear	7
Ahn,[21] 2019	somewhat*	same institute*	record*	no	**	record*	yes*	yes*	8

Table S2: continued.

Study ID	SELECTION				COMPARABILITY	OUTCOME			Total[a]
	Representative-ness of the exposed cohort	Selection of the non-exposed cohort	Ascertainment of exposure	Demonstration that outcome of interest was not present at start of study	Comparability of cohorts on the basis of the design or analysis[b]	Assessment of outcome	Was follow-up long enough for outcomes to occur[c]	Adequacy of follow up of cohorts[d]	
Berner,[20] 2019	somewhat*	same institute*	record*	yes*	-	record*	not clear	not clear	5
Verzoni,[7] 2019	somewhat*	same institute*	record*	no	*	record*	not clear	not clear	5
Yamauchi,[13] 2019	truly*	same institute*	record*	no	-	record*	yes*	not clear	5
Bjørnhart,[28] 2019	somewhat*	same institute*	record*	no	-	record*	not clear	yes*	5
Ishihara,[17] 2019	somewhat*	same institute*	record*	no	*	record*	yes*	not clear	6
Moel,[33] 2019	somewhat*	same institute*	record*	no	-	record*	not clear	not clear	4
Lang,[29] 2019	somewhat*	same institute*	record*	no	-	record*	not clear	not clear	4

- indicates Zero score, * indicates one score, ** indicates two scores.

[a] Each study could be awarded a maximum of nine stars: a maximum of two stars for the item regarding comparability and a maximum of one star for other 7 items.

[b] One score was awarded if a study was a prospective cohort study.

[c] A maximum of two stars could be awarded for this item. If a study performed landmark analysis, one score was awarded. If a study adjusted for confounding factors (eg. ECOG PS, age, metastases status, serum low density lipoprotein level, prior treatment line, etc.), an additional score was awarded.

[d] For studies reporting OS or PFS, if median OS or PFS was reached, one score was awarded. For studies reporting both OS and PFS, only if median OS and PFS were both reached, one score was awarded.

[e] If a study reported a follow up rate of more than or equal to 80%, one score was awarded.

Figure S1. Subgroup analysis stratified by class of immune checkpoint inhibitors in the melanoma cohort.

Source	Sample	HR (95% CI)	% Weight
**Anti−PD−1**			
Keller et al,[9] 2016	143	0.42 (0.24, 0.74)	18.4
Nakamura et al,[22] 2017	35	0.16 (0.03, 0.79)	6.64
Indini et al,[4] 2018	173	0.39 (0.18, 0.81)	15.4
Okada et al,[6] 2019	15	0.01 (0.00, 0.88)	1.16
**Subtotal**	**366**	**0.35 (0.21, 0.61)**	**41.7**
**(I-squared = 19.6%, *p* = 0.292)**			
**Anti−CTLA−4**			
Faje et al,[8] 2018	217	0.53 (0.36, 0.75)	21.2
Moel et al,[33] 2019	133	1.12 (0.70, 1.79)	19.7
Lang et al,[29] 2019	100	1.32 (0.71, 2.44)	17.5
**Subtotal**	**450**	**0.89 (0.49, 1.61)**	**58.3**
**(I-squared = 78.6%, *p* = 0.009)**			
**Overall**	**816**	**0.58 (0.35, 0.95)**	**100**
**(I-squared = 72.3%, *p* < 0.001)**			

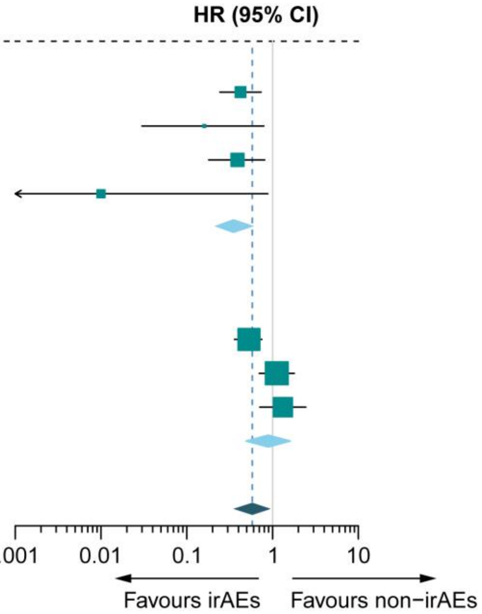

Abbreviations: HR, hazard ration; anti-PD-1, anti-programmed cell death-1; anti-CTLA-4, anti-cytotoxic T-lymphocyte antigen-4; irAEs, immune-related adverse events; non-irAEs, non-immune-related adverse events.

Figure S2. Forest plot (random effects model) of the correlation between immune-related adverse event development and progression-free survival.

Source	Sample	HR (95% CI)	% Weight
Sanlorenzo et al,[32] 2015[a]	43	0.82 (0.17, 4.06)	0.89
Sanlorenzo et al,[32] 2015[b]	24	0.70 (0.05, 9.50)	0.34
Sanlorenzo et al,[32] 2015[c]	16	0.12 (0.02, 0.74)	0.70
Haratani et al,[10] 2017	105	0.54 (0.30, 0.97)	4.02
Kim et al,[11] 2017	58	0.38 (0.17, 0.85)	2.72
Osorio et al,[12] 2017	48	0.58 (0.27, 1.21)	3.00
Nakamura et al,[22] 2017	35	0.24 (0.11, 0.55)	2.72
Grangeon et al,[14] 2018	270	0.42 (0.32, 0.57)	7.21
Toi et al,[18] 2018	137	0.45 (0.30, 0.68)	5.79
Sato et al,[31] 2018	18	0.28 (0.04, 1.46)	0.70
Rogado et al,[25] 2018	106	0.44 (0.28, 0.71)	5.19
Ricciuti et al,[15] 2018	195	0.48 (0.34, 0.67)	6.60
Indini et al,[4] 2018	173	0.47 (0.26, 0.86)	4.00
Lesueur et al,[26] 2018	104	0.66 (0.43, 1.10)	5.18
Lisberg et al,[27] 2018	97	0.62 (0.40, 0.96)	5.48
Fujimoto et al,[30] 2018	613	0.76 (0.55, 1.01)	7.03
Lei et al,[16] 2019	103	0.45 (0.27, 0.76)	4.68
Cortellini et al,[19] 2019	524	0.59 (0.47, 0.76)	7.80
Ahn et al,[21] 2019	111	0.43 (0.26, 0.74)	4.59
Berner et al,[20] 2019	83	0.22 (0.09, 0.39)	3.09
Yamauchi et al,[13] 2019	175	0.66 (0.46, 0.95)	6.32
Bjornhart et al,[28] 2019	112	0.71 (0.39, 1.27)	4.06
Ishihara et al,[17] 2019	47	0.25 (0.11, 0.56)	2.68
Lang et al,[29] 2019	100	1.40 (0.88, 2.22)	5.22
**Overall**	**3297**	**0.52 (0.44, 0.61)**	**100**
**(I-squared = 52.5%, *p* = 0.001)**			

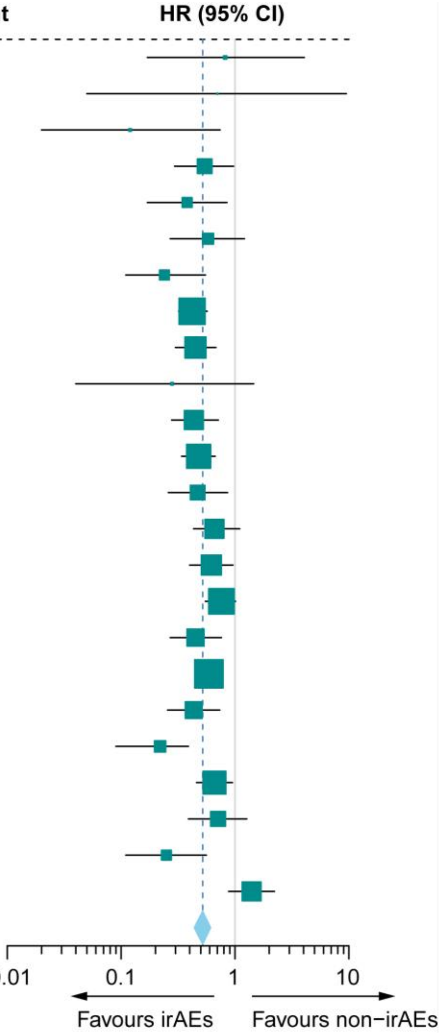

The sizes of the squares indicate the weight of the study. Abbreviations: HR, hazard ratio; irAEs, immune-related adverse events; non-irAEs, non-immune-related adverse events.

[a] The patient group received a dose of 10 mg/kg every 3 weeks.

[b] The patient group received a dose of 10 mg/kg every 2 weeks.

[c] The patient group received a dose of 2 mg/kg every 3 weeks.

Figure S3. Subgroup analyses of the correlation between immune-related adverse event development and progression-free survival.

Subgroup analyses for PFS	No. of studies	Pooled HR (95%CI) Random effect	I^2	Reference
**Patient Characteristics**				
Cancer type				
NSCLC	14	0.53 (0.46-0.61)	32.0%	10-12, 14-15, 18-21, 26-28, 30-31
Melanoma	3	0.56 (0.20-1.57)	88.3%	4, 22, 29
Others [a]	5	0.47 (0.34-0.64)	28.3%	13, 16-17, 25, 32
Combination therapy				
Yes [b]	3	0.77 (0.41-1.45)	78.5%	4, 26, 29
No [c]	19	0.50 (0.43-0.57)	34.4%	10-22, 25, 27-28, 30-32
**Study Quality Characteristics**				
Sample size				
⩾100	14	0.58 (0.48-0.71)	64.9%	4, 10, 13-16, 18-19, 21, 25-26, 28-30
<100	8	0.37 (0.26-0.53)	30.8%	11-12, 17, 20, 22, 27, 31-32
Model				
Multivariate	12	0.54 (0.47-0.63)	16.7%	4, 10-11, 15, 17, 19, 21, 25-27, 30, 32
Univariate	10	0.51 (0.37-0.70)	72.4%	12-14, 16, 18, 20, 22, 28-29, 31
Landmark analysis				
Yes	4	0.55 (0.45-0.68)	0.0%	10, 19, 21, 31
No	18	0.52 (0.43-0.62)	59.3%	4, 11-18, 20, 22, 25-30, 32
Trial design				
Prospective	2	0.36 (0.14-0.92)	69.5%	12, 20
Retrospective	20	0.53 (0.46-0.63)	50.5%	4, 10-11, 13-19, 21-22, 25-32

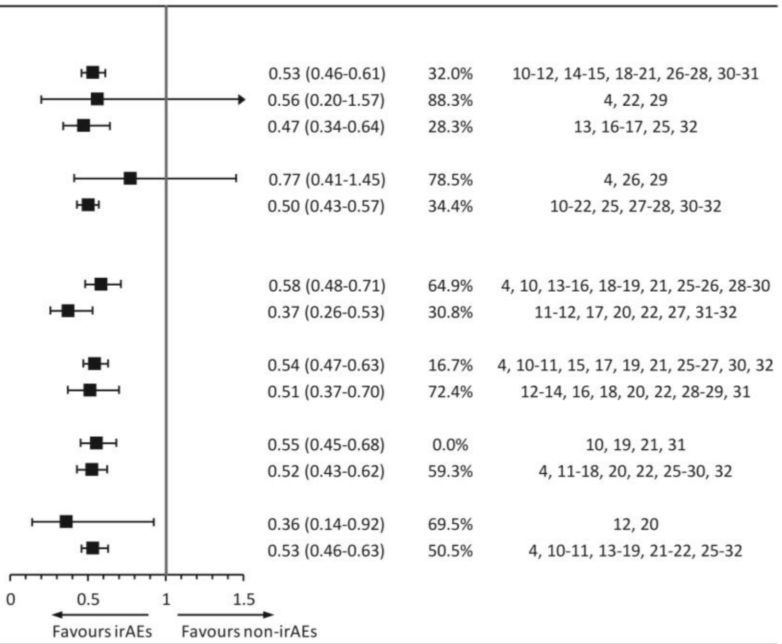

Abbreviations: PFS, progression-free survival; HR, hazard ratio; NSCLC, non-small cell lung cancer; irAEs, immune-related adverse events; non-irAEs, non-immune-related adverse events.

[a] This group included 4 multiple cancer types and 1 renal cell carcinoma.

[b] Yes indicates studies that combined ICIs with other therapy, including peptide vaccine (n=1), radiotherapy (n=1) and Vemurafenib (n=1).

[c] No indicates studies that adopted ICIs as monotherapy.

Figure S4. Sensitivity analysis of the impact of each individual study on the pooled effect. A) Overall survival; B) Progression-free survival.

A

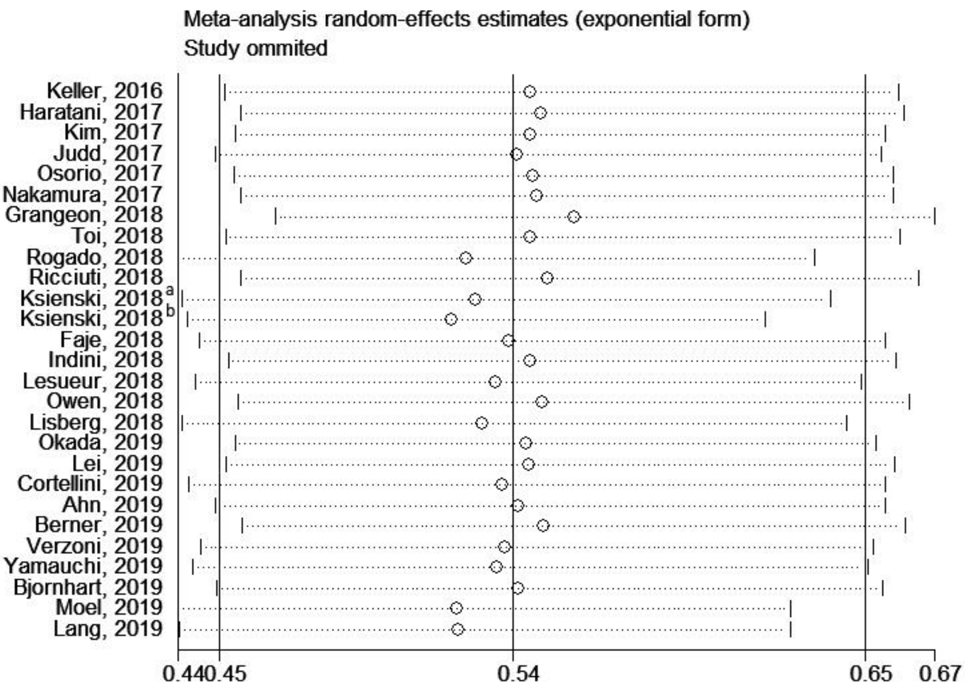

B

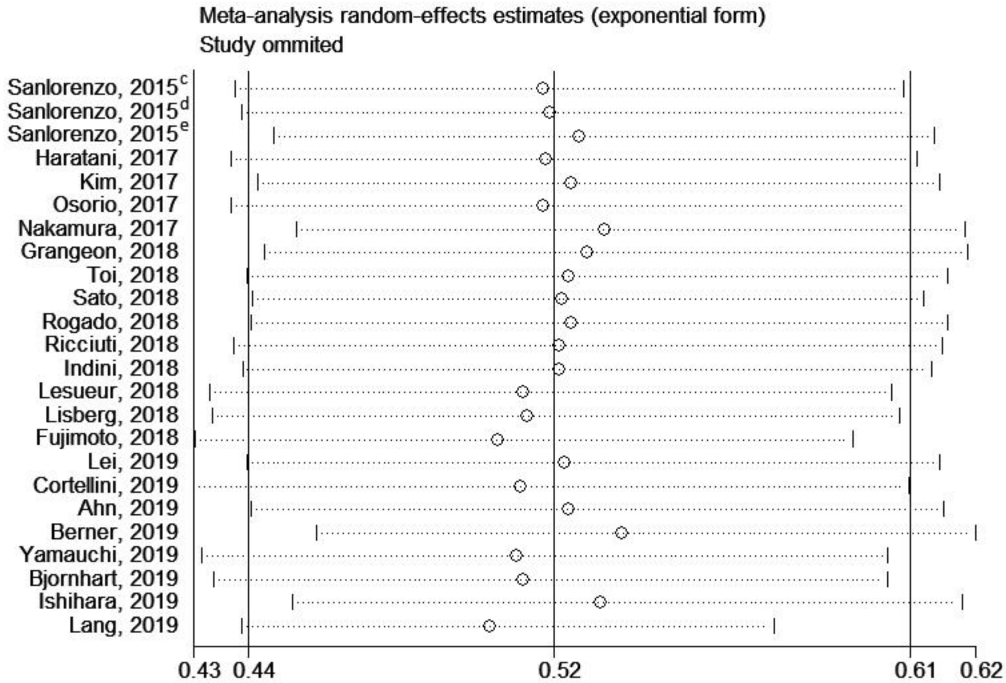

[a] Result for grade 1-2 immune-related adverse events (irAEs).

[b] Result for grade 3-4 irAEs.

[c] The patient group received a dose of 10 mg/kg every 3 weeks.

[d] The patient group received a dose of 10 mg/kg every 2 weeks.

[e] The patient group received a dose of 2 mg/kg every 3 weeks.

Figure S5. Funnel plots of the overall survival results. (A) Without trim and fill; (B) With trim and fill.

A

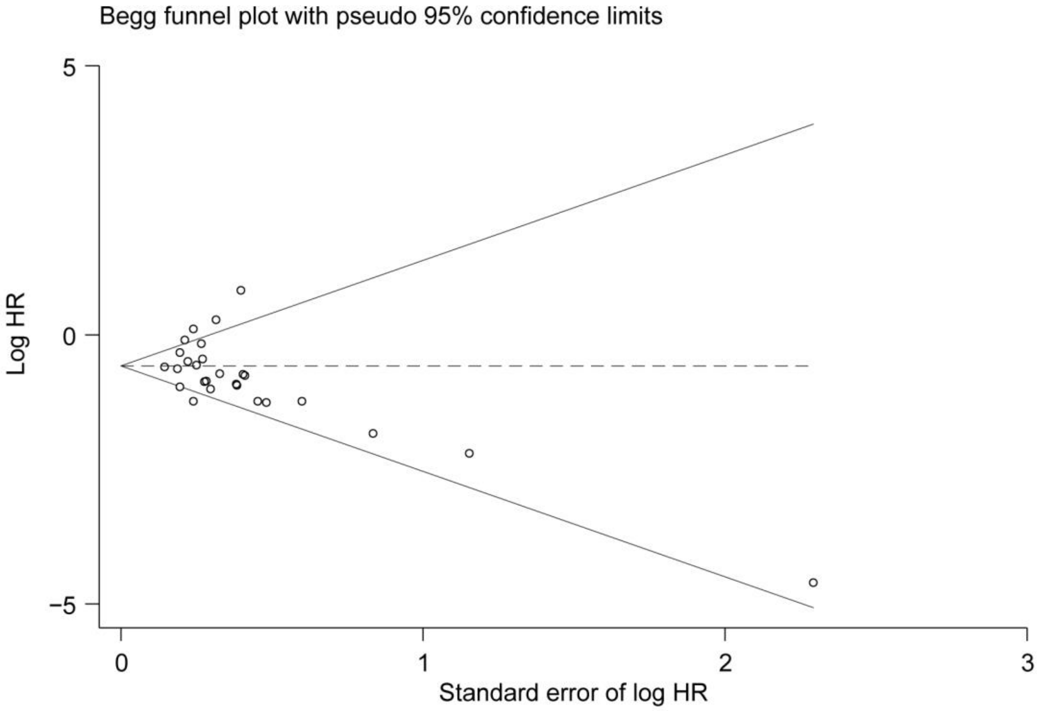

B

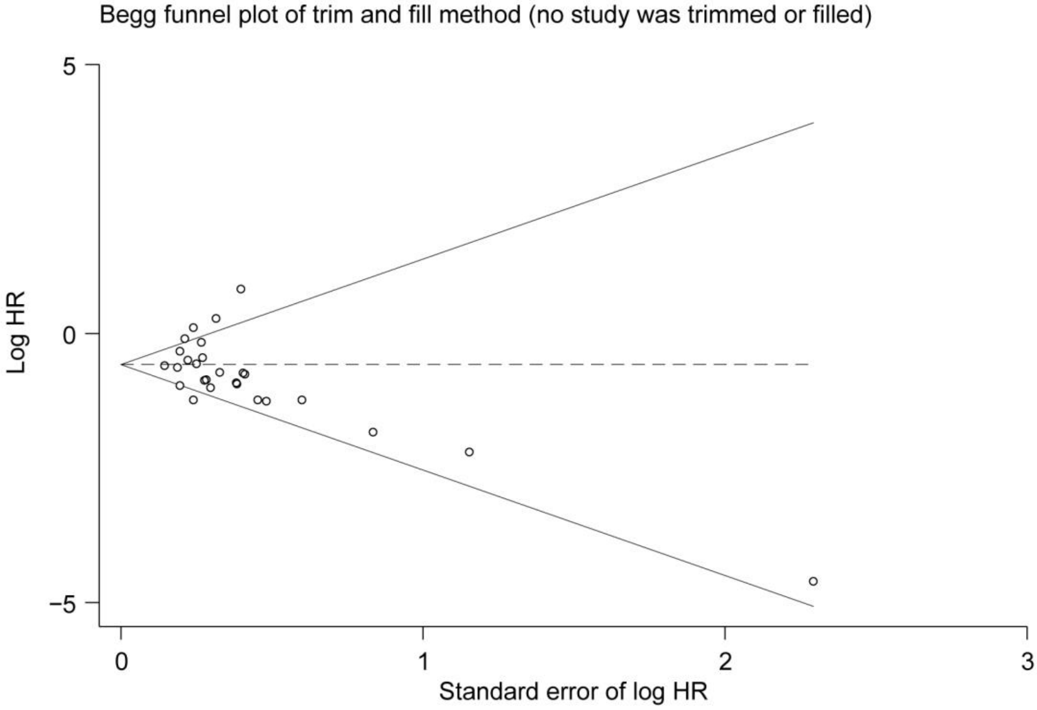

Abbreviation: HR, hazard ratio.

Figure S6. Funnel plots of the overall survival results in large sample size studies.

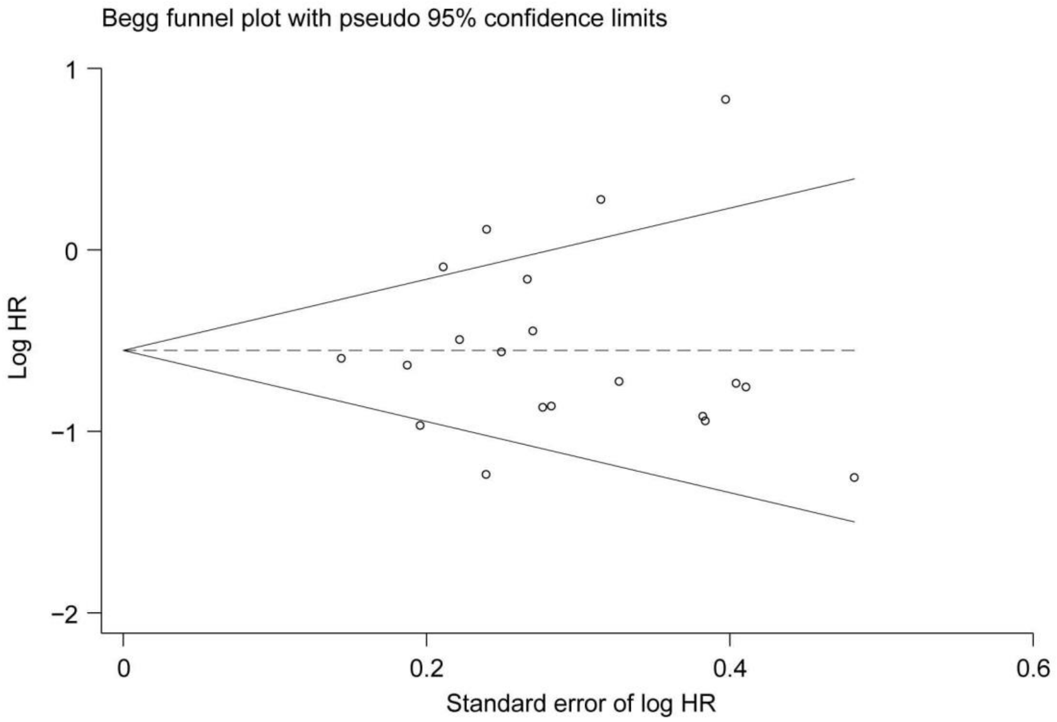

Abbreviation: HR, hazard ratio.

Figure S7. Funnel plots of the progression-free survival results.

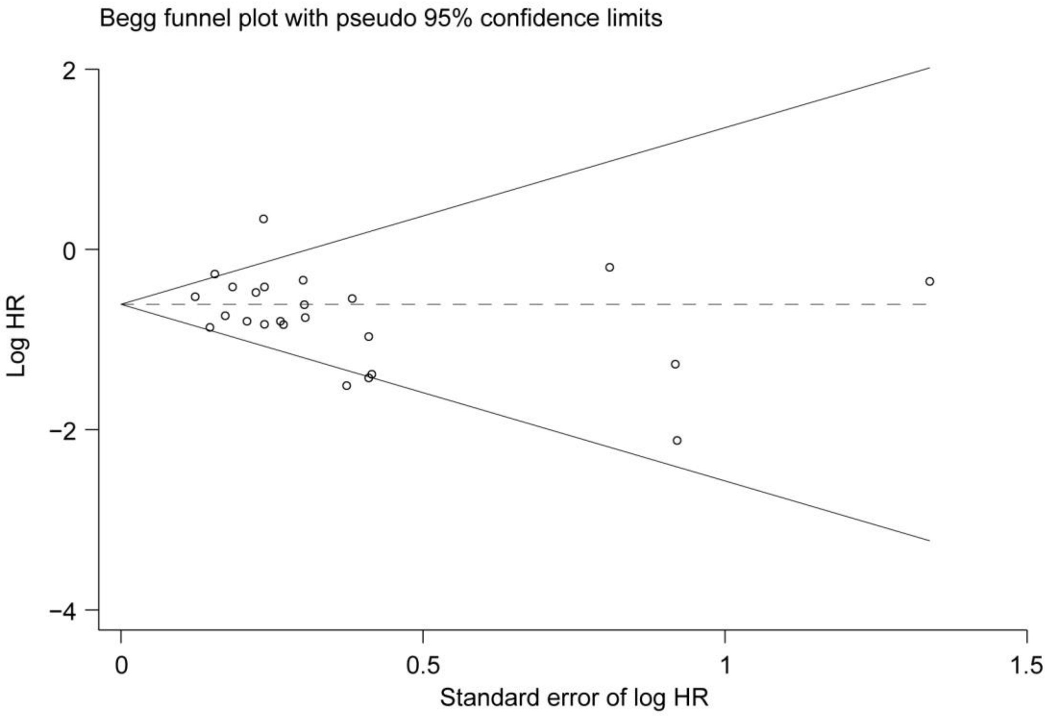

Abbreviation: HR, hazard ratio.
