# Supplementary material for: Are immune-related adverse events associated with the efficacy of immune checkpoint inhibitors in patients with cancer? A systematic review and meta-analysis
Source: BMC Med. 2020 Apr 20;18:87. doi: 10.1186/s12916-020-01549-2 (PMC7169020; doi:10.1186/s12916-020-01549-2)
Supplement: Supplementary file 2 — Log file of trim and fill method in Figure S5. [file 12916_2020_1549_MOESM2_ESM.pdf]

Statistics/Data Analysis

```
      name:  <unnamed>
       log:  C:\Users\Zhou Xiaoxiang\Downloads\Stata12.0\Trim_and_fill_record.smcl
  log type:  smcl
 opened on:   7 Dec 2019, 09:08:39

1 . use "C:\Users\Zhou Xiaoxiang\Desktop\Any OS.dta", clear

2 . metan log_HR log_down log_upper, label(namevar=Study) random eform

           Study     |     ES    [95% Conf. Interval]     % Weight
---------------------+---------------------------------------------------
Keller, 2016         |  0.423       0.243     0.735          4.26
Haratani, 2017       |  0.285       0.102     0.675          2.46
Kim, 2017            |  0.110       0.010     0.920          0.61
Judd, 2017           |  0.480       0.227     1.107          3.04
Osorio, 2017         |  0.290       0.090     0.940          1.82
Nakamura, 2017       |  0.160       0.030     0.790          1.08
Grangeon, 2018       |  0.290       0.180     0.460          4.78
Toi, 2018            |  0.420       0.240     0.710          4.33
Rogado, 2018         |  0.909       0.625     1.429          5.13
Ricciuti, 2018       |  0.380       0.260     0.560          5.32
Ksienski, 2018       |  0.850       0.500     1.420          4.45
Ksienski, 2018       |  2.290       1.050     4.980          3.10
Faje, 2018           |  0.530       0.360     0.750          5.43
Indini, 2018         |  0.390       0.180     0.810          3.22
Lesueur, 2018        |  0.640       0.377     1.087          4.41
Owen, 2018           |  0.364       0.203     0.649          4.10
Lisberg, 2018        |  0.720       0.490     1.050          5.34
Okada, 2019          |  0.010       0.000     0.880          0.16
Lei, 2019            |  0.400       0.190     0.850          3.23
Cortellini, 2019     |  0.550       0.410     0.720          5.95
Ahn, 2019            |  0.484       0.255     0.919          3.77
Berner, 2019         |  0.290       0.120     0.710          2.66
Verzoni, 2019        |  0.570       0.350     0.930          4.66
Yamauchi, 2019       |  0.610       0.390     0.930          5.00
Bjornhart, 2019      |  0.470       0.210     1.050          2.99
Moel, 2019           |  1.120       0.700     1.790          4.78
Lang, 2019           |  1.320       0.710     2.440          3.90
---------------------+---------------------------------------------------
D+L pooled ES        |  0.541       0.450     0.651        100.00
---------------------+---------------------------------------------------
 Heterogeneity calculated by formula
  Q = SIGMA_i{ (1/variance_i)*(effect_i - effect_pooled)^2 }
where variance_i = ((upper limit - lower limit)/(2*z))^2

  Heterogeneity chi-squared =  68.72 (d.f. = 26) p = 0.000
  I-squared (variation in ES attributable to heterogeneity) =  62.2%
  Estimate of between-study variance Tau-squared =  0.1285

  Test of ES=1 : z=   6.52 p = 0.000

3 . metatrim log_HR selog_HR, reffect eform funnel

Note: default data input format (theta, se_theta) assumed.

Meta-analysis

       |  Pooled      95% CI         Asymptotic      No. of
Method |     Est   Lower   Upper  z_value  p_value   studies
-------+----------------------------------------------------
Fixed  |  -0.577  -0.682  -0.473  -10.862    0.000     27
Random |  -0.614  -0.799  -0.429   -6.516    0.000

Test for heterogeneity: Q= 68.723 on 26 degrees of freedom (p= 0.000)
Moment-based estimate of between studies variance =  0.129

Trimming estimator: Linear
Meta-analysis type: Random-effects model
```

```
iteration |  estimate    Tn    # to trim     diff
----------+--------------------------------------
    1     |   -0.614    131         0         378
    2     |   -0.614    131         0           0

Note: no trimming performed; data unchanged

Filled
Meta-analysis (exponential form)

       |  Pooled      95% CI         Asymptotic      No. of
Method |     Est   Lower   Upper  z_value  p_value   studies
-------+----------------------------------------------------
Fixed  |   0.561   0.506   0.623  -10.862    0.000     27
Random |   0.541   0.450   0.651   -6.516    0.000

Test for heterogeneity: Q= 68.723 on 26 degrees of freedom (p= 0.000)
Moment-based estimate of between studies variance =  0.129

4 . log close
      name:  <unnamed>
       log:  C:\Users\Zhou Xiaoxiang\Downloads\Stata12.0\Trim_and_fill_record.smcl
  log type:  smcl
 closed on:   7 Dec 2019, 09:09:58
```
